# Belimumab concentrations and immunogenicity in relation to drug effectiveness and safety in SLE within a Swedish real-world setting

**DOI:** 10.1093/rheumatology/keaf128

**Published:** 2025-03-03

**Authors:** Alvaro Gomez, Tomas Walhelm, Floris C Loeff, Andreas Jönsen, Dionysis Nikolopoulos, Bryan van den Broek, Anders A Bengtsson, Annick de Vries, Theo Rispens, Christopher Sjöwall, Ioannis Parodis

**Affiliations:** Division of Rheumatology, Department of Medicine Solna, Karolinska Institutet, Karolinska University Hospital, and Center for Molecular Medicine (CMM), Stockholm, Sweden; Division of Inflammation and Infection/Rheumatology, Department of Biomedical and Clinical Sciences, Linköping University, Linköping, Sweden; R&D, Sanquin Diagnostic Services, Amsterdam, The Netherlands; Department of Clinical Sciences Lund, Rheumatology, Lund University, Skåne University Hospital, Lund, Sweden; Division of Rheumatology, Department of Medicine Solna, Karolinska Institutet, Karolinska University Hospital, and Center for Molecular Medicine (CMM), Stockholm, Sweden; R&D, Sanquin Diagnostic Services, Amsterdam, The Netherlands; Department of Clinical Sciences Lund, Rheumatology, Lund University, Skåne University Hospital, Lund, Sweden; R&D, Sanquin Diagnostic Services, Amsterdam, The Netherlands; R&D, Sanquin Diagnostic Services, Amsterdam, The Netherlands; Amsterdam Institute for Infection and Immunity, Amsterdam University Medical Center, Amsterdam, The Netherlands; Department of Immunopathology, Sanquin Research, Amsterdam, The Netherlands; Molecular Cell Biology and Immunology, Amsterdam UMC location Vrije Universiteit Amsterdam, Amsterdam, The Netherlands; Division of Inflammation and Infection/Rheumatology, Department of Biomedical and Clinical Sciences, Linköping University, Linköping, Sweden; Division of Rheumatology, Department of Medicine Solna, Karolinska Institutet, Karolinska University Hospital, and Center for Molecular Medicine (CMM), Stockholm, Sweden; Department of Rheumatology, Faculty of Medicine and Health, Örebro University, Örebro, Sweden

**Keywords:** systemic lupus erythematosus, belimumab, anti-drug antibodies, immunogenicity, B cells, B lymphocyte, biologics, therapeutic monitoring

## Abstract

**Objectives:**

Studies supporting therapeutic drug monitoring to biopharmaceuticals in SLE are scarce. We aimed to assess anti-drug antibody (ADA) occurrence in belimumab-treated SLE patients and associations between belimumab concentrations and clinical response, serological outcomes and adverse events.

**Methods:**

We included 100 patients treated with intravenous belimumab. Clinical data and biological samples were collected at baseline and months 3, 6, 12 and 24. Belimumab levels were determined by quantitative sandwich ELISA, and ADA by an acid-dissociation radioimmunoassay. Clinical activity was evaluated with the SLEDAI-2000 (SLEDAI-2K), revised SLE activity measure (SLAM-R) and physician’s global assessment (PhGA). Serological markers included C3, C4 and anti-dsDNA. We performed cross-sectional Spearman’s rank correlation analyses, and longitudinal analyses using generalized estimating equations.

**Results:**

Belimumab concentrations varied widely (median: 25.8; interquartile range [IQR]: 20.9–43.5 μg/ml) but were stable over time at the group level. Pre-existing ADA was detected in two patients, but no patient developed ADA during follow-up. Belimumab levels moderately correlated with SLEDAI-2K (*ρ*: −0.37; *P* = 0.003) and PhGA (*ρ*: −0.41; *P* = 0.005) at month 6, while longitudinal analysis revealed a very weak association with SLEDAI-2K (*β*: −0.10; SE: 0.05; *P* = 0.031) and a weak association with SLAM-R (*β*: −0.32; SE: 0.13; *P* = 0.014). Despite moderate correlations between belimumab levels and serological markers at month 6, there were no associations in longitudinal analysis. There was no relationship between belimumab levels and adverse events.

**Conclusion:**

Belimumab yielded no immunogenicity. Belimumab levels were modestly associated with clinical activity but not with serological activity or adverse events.

Rheumatology key messages

- Belimumab showed no immunogenicity; anti-drug antibodies are unlikely contributors to belimumab inefficacy or adverse events.
- Belimumab concentrations showed inter- and intra-individual variations but consistent group-level distributions across study visits.
- Intravenous belimumab at doses lower than approved may maintain benefits, suggesting potential for dosing optimization.

## Introduction

Belimumab is a human monoclonal antibody that targets soluble B cell activating factor belonging to the TNF family (BAFF), also known as B lymphocyte stimulator (BLyS), through which it induces dynamic changes in various B cell subsets [1, 2]. Belimumab is licensed for the treatment of moderately active, autoantibody positive SLE and active lupus nephritis [3, 4].

During the past two decades, belimumab has shown efficacy and a favourable safety profile in several randomized controlled trials (RCTs) and observational studies [5]. However, a substantial proportion of patients fail to achieve desired outcomes with belimumab treatment, as reported using the SLE Response Index (SRI)-4 (responder frequencies: 43–61%) [6–10], lupus low disease activity state (LLDAS) (attainer frequencies: 17–63%) [11, 12] and remission (attainer frequencies: 8–36%) after 1 year of treatment [11, 12]. As evidenced in SLE for the use of rituximab [13] and in other immune-mediated diseases for several biopharmaceuticals [14–16], a potential explanation for the lack of efficacy is low drug bioavailability, possibly as a consequence of anti-drug antibody (ADA) formation. Within rheumatology, most evidence derives from TNF inhibitors in patients with inflammatory arthropathies, with several studies showing associations between low drug levels and poor treatment outcomes, including higher disease activity [14].

Methods to quantify belimumab concentrations have become available [17, 18]. To date, such assays have been employed to study associations between drug levels and adherence to belimumab administered subcutaneously [19] and drug transfer to breast milk [20]. However, associations between belimumab concentrations and clinical efficacy, as well as the occurrence and potential impact of ADA against belimumab, remain unknown. In this study, we aimed to determine the incidence and levels of ADA in SLE patients treated with intravenous belimumab and to investigate the association between belimumab concentrations and clinical outcome, serological response and adverse events over time on treatment.

## Methods

### Study population

Patients with SLE meeting the 1982 ACR classification criteria [21] and/or 2012 SLICC criteria [22] and treated with belimumab have been followed prospectively since 2011 at the Karolinska, Linköping and Skåne University Hospitals in Sweden [23, 24]. Clinical and routine laboratory parameters, patient-reported data and biological samples have been collected at baseline, month 3, month 6, month 12 and yearly thereafter, or more frequently if clinically indicated. The population for the present investigation was restricted to individuals who received intravenous belimumab, provided serum samples and have been followed for at least two visits, unless belimumab was discontinued early (before a second visit was scheduled) due to an adverse event.

All study participants provided written informed consent for participation in the study. Ethical permission was granted by the Swedish Ethical Review Authority (2023–00938-02) and the Regional Ethics Board in Linköping (M75-08) and Lund (LU378-02).

### Measurement of belimumab levels and anti-drug antibodies

Serum samples were processed and stored at −80°C at each participating centre. Belimumab and ADA levels were determined at Sanquin Diagnostic Services, Amsterdam, the Netherlands. Belimumab levels were measured using a quantitative sandwich ELISA, as recently described [17]. Briefly, rabbit anti-idiotype antibodies were used to capture belimumab, and anti-belimumab biotinylated F(ab’)2 fragments were used to detect the complex. The validated reportable range of the assay is 0.03–625 μg/ml.

Aiming to minimize non-random variability due to the timing of sampling, serum samples were typically obtained on the same day as the clinical evaluation, which often coincided with the day of infusion. Moreover, samples were typically drawn before the infusion. We excluded non-trough samples collected up to 14 days post infusion (23/350) from the analysis of belimumab levels (Supplementary Fig. S1, available at *Rheumatology* online).

ADA against belimumab was measured in all samples, independent of drug levels and time of sampling, using a drug-tolerant acid-dissociation radioimmunoassay (ARIA), which is able to detect ADA even in the presence of high drug levels [25]. The lower limit of detection was set at 15.2 AU/ml, corresponding to mean level plus three standard deviations in samples from 35 healthy individuals, confirmed using samples obtained prior to belimumab exposure from the Swedish belimumab cohort.

### Study outcomes: measures of clinical and serological response, and adverse events

Disease activity was assessed at every visit with the SLEDAI-2000 (SLEDAI-2K), a widely used instrument comprising 24 descriptors grouped into nine organ systems [26], and the Physician’s Global Assessment (PhGA; 0–100 mm) in all centres. In patients from the Karolinska University Hospital, disease activity was additionally evaluated with the revised version of SLAM (SLAM-R) [27], which includes 31 items grouped into 10 organ systems, but excludes serological activity. Complement C3 and C4 levels were quantified using nephelometry; low C3 was defined as levels < 0.67 g/l and low C4 as levels < 0.13 g/l. Anti-dsDNA titres were determined using the *Crithidia luciliae* immunofluorescence test (CLIFT), with a positivity cut-off at a titre of 1:10. Adverse events were documented at each visit by the treating physician and supplemented with chart reviews.

### Other variables

We collected patient characteristics at baseline, including age, sex, disease duration and use of antimalarial agents, glucocorticoids and immunosuppressants. Furthermore, organ damage was assessed at every visit using the SLICC/ACR Damage Index (SDI) [28].

### Statistical analysis

Continuous variables were summarized using medians and interquartile range (IQR), and categorical variables using frequencies (percentage). To evaluate the variation of belimumab levels over time on treatment, we used linear mixed models, including the patients as a random effect in the models. We compared a nested model with a fixed effect for time (visit) with a model without this effect using a likelihood ratio test.

For cross-sectional analyses of continuous outcomes (i.e. SLEDAI-2K score, SLAM-R score, PhGA score, C3 levels, C4 levels and anti-dsDNA titres), we estimated correlations with belimumab concentrations at month 3, month 6 and month 12 using Spearman’s correlation coefficients (*ρ*). In addition, we compared the distributions of continuous outcomes across belimumab quartiles for the same time points using the Mann–Whitney U test, with the lowest quartile as the reference group. To account for differences across laboratories, analyses of anti-dsDNA titres were conducted with data from Karolinska University Hospital only. Analyses of anti-dsDNA positivity were conducted using data from all centres.

For cross-sectional analyses of binary outcomes (i.e. low *vs* normal/high C3, low *vs* normal/high C4, positive *vs* negative anti-dsDNA antibody titres, and occurrence *vs* absence of adverse events), we compared the distributions of belimumab concentrations between categories. For longitudinal analyses, we employed generalized estimating equations (GEE), using data for all available assessments and assuming an independent correlation matrix, with robust standard errors. For ease of interpretation, belimumab concentrations were scaled in the models so that the estimates correspond to the effect of a 10 μg/ml difference in belimumab levels. Models were adjusted for age, sex and baseline values for the outcome of interest. As there were no missing data for covariates in the GEE models, we conducted complete case analyses, with no data imputation performed.

Given the minimal occurrence of ADA development, we did not estimate measures of associations. Instead, we described the clinical course of the two patients who developed ADA during the study period.

Data management and statistical analyses were performed using R version 4.3.1 (R Foundation for Statistical Computing, Vienna, Austria). The level of statistical significance was set at 5%.

### Patient and public involvement

Patients and/or the public were not involved in the design, conduct, reporting or dissemination plans of this research.

## Results

### Description of the study population

The study population included 100 individuals receiving at least one belimumab infusion, whose characteristics are summarized in Table 1. Based on the ACR classification criteria, ≥85% of patients had presented with mucocutaneous, musculoskeletal or haematological manifestations until baseline, and a third of them with renal disease. At baseline, the median SLEDAI-2K score was 8.0 (4.0–12.0) and the median PhGA score was 50 (31–67) mm, while nearly half of the patients had low C3 or C4 and/or positive anti-dsDNA antibodies. The median disease duration was 8 (4–19) years, and 44% of the patients had developed organ damage at the baseline evaluation prior to belimumab initiation. Most patients were receiving antimalarial agents (81%), glucocorticoids (92%) and at least one immunosuppressant (65%) at baseline.

**Table 1.** Demographics and clinical characteristics of study participants at baseline

	Population (*N* = 100)
Age at baseline (years), median (IQR)	41.7 (32.2–51.3)
Female sex, *n* (%)	89 (89.0)
Site, *n* (%)	
Karolinska University Hospital	71 (71.0)
Linköping University Hospital	12 (12.0)
Skåne University Hospital	17 (17.0)
Ethnic origin, *n* (%); *N* = 91	
Asian	6 (6.6)
Black/African American	6 (6.6)
White	79 (86.8)
Disease duration (years), median (IQR)	8.2 (3.7–18.9)
ACR criteria, *n* (%)	
Malar rash	69 (69.0)
Discoid rash	29 (29.0)
Photosensitivity	56 (56.0)
Mucosal ulcers	58 (58.0)
Arthritis	86 (86.0)
Serositis	31 (31.0)
Renal disease	33 (33.0)
Neuropsychiatric disease	10 (10.0)
Haematologic disease	85 (85.0)
Immunological	80 (80.0)
ANA	100 (100)
SLEDAI-2K score, median (IQR)	8.0 (4.0–10.0)
PhGA (mm), median (IQR); *N* = 70	50.0 (31.0–67.0)
SLAM-R score, median (IQR); *N* = 67	10.0 (7.0–16.0)
SDI score, median (IQR)	0 (0–1)
SDI score > 0, *n* (%)	44 (44.0)
C3 level (g/l), median (IQR)	0.81 (0.64–1.01)
Low C3, *n* (%)	43 (43.0)
C4 level (g/l), median (IQR)	0.13 (0.08–0.18)
Low C4, *n* (%)	49 (49.0)
Anti-dsDNA titre, median (IQR)	10 (0–100)
Anti-dsDNA (+), *n* (%)	48 (48.0)
Medications until baseline, *n* (%)	
Antimalarial agents	100 (100)
Glucocorticoids	100 (100)
Azathioprine	77 (77.0)
Methotrexate	48 (48.0)
Mycophenolic acid	48 (48.0)
Medications at baseline, *n* (%)	
Antimalarial agents	81 (81.0)
Glucocorticoids	92 (92.0)
Current prednisolone dose (mg/day), median (IQR)	10.0 (5.00–12.5)
Azathioprine	28 (28.0)
Methotrexate	15 (15.0)
Mycophenolic acid	17 (17.0)

The number of observations is indicated when it differed from the total population. IQR: interquartile range; PhGA: physician’s global assessment; SDI: SLICC/ACR damage index; SLAM-R: revised SLAM; SLEDAI-2K: SLEDAI-2000.

As shown in Supplementary Fig. S2, available at *Rheumatology* online, 49% of the patients received intravenous belimumab for at least 24 months. The most common reasons for treatment discontinuation were lack of effectiveness (*n* = 21) and adverse events (*n* = 7). Lack of effectiveness was deemed to be primary in all cases; the mean time from baseline until discontinuation due to lack of effectiveness was 10.1 ± 4.7 months. Eight individuals switched to subcutaneous form before 24 months, and did not contribute with samples after switching.

### Description of serum levels of belimumab over time

**Figure 1.**
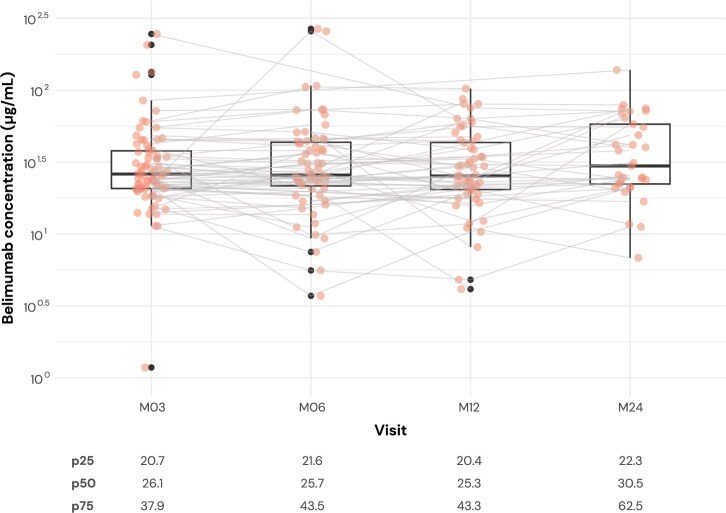
Belimumab serum concentration during follow-up. Distribution of serum belimumab levels at each visit for the total cohort. Orange circles denote individual measurements. Boxplots represent percentiles (10th, 25th, 50th, 75th and 90th), while dark dots represent outliers. Grey lines connect belimumab levels within individual patients

The median trough serum concentration of belimumab was 25.8 μg/ml (IQR: 20.9–43.5 μg/ml; range: 1.2–366.8 μg/ml) in all samples obtained from month 3 until month 24 after treatment initiation. The corresponding median levels over time were 26.1 μg/ml (IQR: 20.7–37.9 μg/ml) at month 3, 25.7 μg/ml (IQR: 21.6–43.5 μg/ml) at month 6, 25.3 μg/ml (IQR: 20.4–43.3 μg/ml) at month 12 and 30.5 μg/ml (IQR: 22.3–62.5 μg/ml) at month 24. The distribution of belimumab concentrations was stable across visits, with a slight increase at month 24 (Fig. 1), which was confirmed by a likelihood ratio test comparing linear mixed models with and without a fixed effect for time (*P* = 0.290).

### Associations between belimumab levels and clinical activity

**Figure 2.**
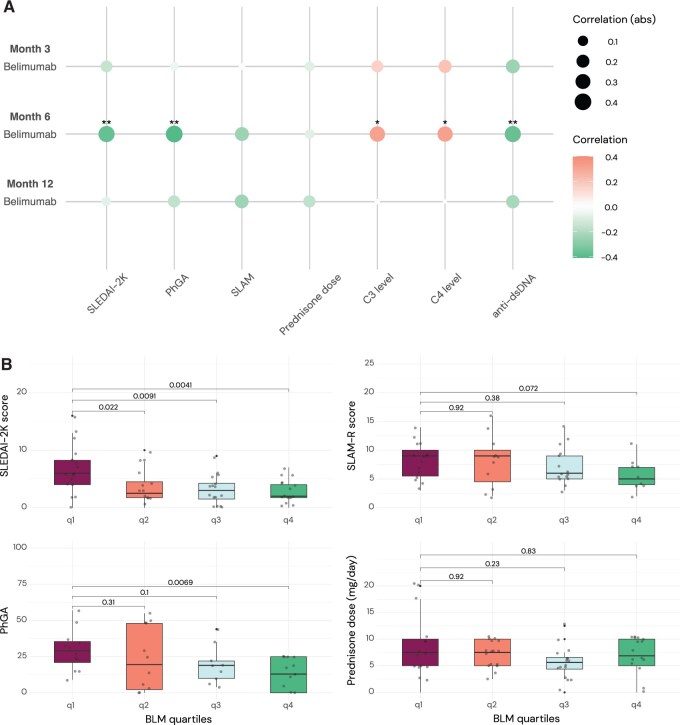
Associations between belimumab serum concentration and measures of clinical and serological activity. (**A**) Spearman’s rank correlation coefficients for cross-sectional analysis between belimumab levels and clinical and serological measures at month 3, month 6 and month 12 after treatment initiation. *P < 0.05. **P < 0.01. (**B**) Comparisons of disease activity measures by quartile of belimumab drug levels at month 6. BLM: belimumab; PhGA: physician’s global assessment; SLAM-R: revised SLAM; SLEDAI-2K: SLEDAI-2000

In cross-sectional analyses, there were moderate inverse correlations between belimumab concentrations and disease activity scores at month 6 (Fig. 2); these were statistically significant for SLEDAI-2K (*ρ*: −0.37; *P* = 0.003) and PhGA (*ρ*: −0.41; *P* = 0.005), but not for SLAM-R (*ρ*: −0.25; *P* = 0.086). Correlations at months 3 and 12 were weaker than those at month 6 for SLEDAI-2K (*ρ*: −0.15 and −0.07), PhGA (*ρ*: −0.06 and −0.16) and SLAM-R (*ρ*: 0.02 and −0.24), respectively. Similarly, at month 6, patients belonging to the lowest belimumab quartile had significantly higher SLEDAI-2K scores (median: 6.0; IQR: 4.0–8.3) than those belonging to the other quartiles (q2: median: 2.5; IQR: 1.8–4.5; *P* = 0.022; q3: median: 3.0; IQR: 1.5–4.3; *P* = 0.009; q4: median: 2.0; IQR: 1.8–4.0; *P* = 0.004), as well as significantly higher PhGA scores than those of quartile 4 (q1: median: 29; IQR: 21–36 *vs* q4: median: 13; IQR: 0–25; *P* = 0.007; Fig. 2).

In longitudinal analyses, higher belimumab concentrations were associated with lower levels of disease activity (Table 2). In adjusted GEE models, these associations were significant, albeit weak, for SLEDAI-2K (*β*: −0.10; SE: 0.05; *P* = 0.031) and SLAM-R (*β*: −0.32; SE: 0.13; *P* = 0.014), but not for PhGA (*β*: −0.31; SE: 0.17; *P* = 0.064).

**Table 2.** Associations of belimumab concentrations with clinical and serological activity measures in longitudinal analyses

	Unadjusted models		Adjusted models	
	*β* (95% CI)	*P* value	*β* (95% CI)	*P* value
SLEDAI-2K	−0.14 (−0.23 to −0.05)	0.002	−0.10 (−0.20 to −0.01)	0.031
PhGA	−0.86 (−1.22 to −0.50)	<0.001	−0.31 (−0.63 to 0.02)	0.064
SLAM-R	−0.29 (−0.45 to −0.13)	<0.001	−0.32 (−0.57 to −0.06)	0.014
Prednisone dose (mg/day)	−0.16 (−0.25 to −0.06)	0.001	−0.11 (−0.20 to −0.02)	0.016
C3 (mg/dl)	0.76 (0.01–1.51)	0.046	0.12 (−0.32 to 0.55)	0.600
C4 (mg/dl)	0.22 (0.00–0.43)	0.047	0.02 (−0.10 to 0.15)	0.710
Anti-dsDNA titre	−8.26 (−26.5 to 9.95)	0.374	2.16 (−10.6 to 14.9)	0.740

Generalized estimating equation models for the association between measured belimumab levels and continuous measures of clinical and serological activity. The estimates correspond to the effect of differences in 10 μg/ml in belimumab concentrations. Covariates in adjusted models: age, sex and baseline values for the outcome of interest. PhGA: physician’s global assessment; SLAM-R: revised SLAM; SLEDAI-2K: SLEDAI-2000.

### Associations between belimumab levels and serological activity

In cross-sectional analyses, there were moderate positive correlations between belimumab concentrations and complement levels at month 6 (*ρ*: 0.31; *P* = 0.014 for C3; and *ρ*: 0.32; *P* = 0.012 for C4; Fig. 1). Likewise, patients with low C3 levels at month 6 had lower belimumab levels (median: 23.6; IQR: 17.8–31.1) than patients with normal C3 levels (median: 33.4; IQR: 23.2–45.4; *P* = 0.039), as did patients with low C4 levels (median: 23.6; IQR: 14.3–29.2) compared with those with normal C4 levels (median: 31.7; IQR: 23.3–44.3; *P* = 0.019; Supplementary Fig. S3, available at *Rheumatology* online). Furthermore, we observed inverse correlations with anti-dsDNA antibody titres at month 6 (*ρ*: −0.37; *P* = 0.008).

In longitudinal analysis, we did not observe strong associations between belimumab concentrations and complement levels or anti-dsDNA antibody titres. All associations attenuated in adjusted GEE models and were non-significant (Table 2).

### Associations between belimumab levels and adverse events

**Figure 3.**
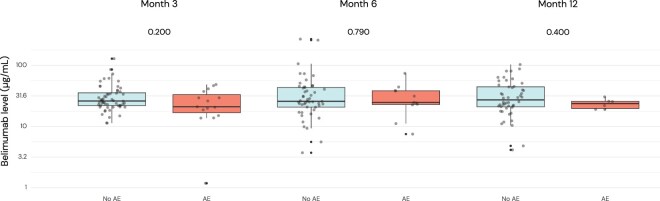
Comparisons of belimumab drug levels by occurrence of adverse events throughout follow-up. Distribution of serum belimumab levels at each visit stratified by occurrence of adverse events. Grey circles denote individual measurements, while boxplots represent percentiles 10th, 25th, 50th, 75th and 90th. AE: adverse event

Fifty-three adverse events (15.1% of visits) were recorded in 36 patients (Supplementary Table S1, available at *Rheumatology* online), resulting in treatment discontinuation in 13 patients and reduction of the dose of belimumab in 4 patients. As shown in Fig. 3, there was no significant difference in belimumab concentrations between patients who developed and those who did not develop adverse events at any time point. The age- and sex-adjusted OR of adverse events for belimumab concentration in longitudinal analysis was 0.94 (95% CI: 0.85–1.04; *P* = 0.221).

### Occurrence of anti-belimumab antibodies

ADA against belimumab was determined in all samples, including baseline samples (Supplementary Fig. S1, available at *Rheumatology* online). Of 350 samples analysed, 3 (0.9%) had ADA levels slightly above the lower limit of detection of the assay, corresponding to 2 patients (Table 3). In patient #1, ADA was detected only at baseline, i.e. prior to belimumab exposure (16.6 AU/ml; samples available: baseline, month 3, month 6, month 12), whereas in patient #2, ADA was detected at baseline (39.6 AU/ml) and month 3 (32.3 AU/ml; samples available: baseline, month 3). Both patients had low belimumab concentrations at month 3 (15.0 μg/ml for patient #1 and 13.8 μg/ml for patient #2, below the threshold of 20.7 μg/ml for the lowest concentration quartile) and developed non-serious adverse events by month 3 (patient #1 developed nausea and malaise after belimumab infusion, and patient #2 developed a mild oral infection), which resolved without modifying the belimumab dosage. Both patients completed 24 months on belimumab treatment and experienced clinical and serological improvement, including improvements in SLEDAI-2K, PhGA and complement levels.

**Table 3.** Clinical course and belimumab concentrations in the two patients with pre-existing ADA against belimumab

	M0	M3	M6	M12	M24
Patient #1					
Sampling	✓	✓	✓	✓	
BEL level (μg/ml)	0	15.03	38.45	19.00	
ADA (AU/ml)^a^	16.55	0	0	0	
SLEDAI-2K	6	4	0	0	0
PhGA	24		22	17	18
SLAM-R	6	4	6	5	3
Prednisolone dose (mg/day)	10	10	7.5	5	5
Anti-dsDNA	0	0	0	0	0
C3 (g/l)	1.19	0.57	1.00	1.21	1.34
C4 (g/l)	0.20	0.09	0.21	0.23	0.26
Adverse event	Nausea	Self-limiting malaise	None	None	None
Patient #2					
Sampling	✓	✓			
BEL level (μg/ml)	0	13.77			
ADA (AU/ml)^a^	39.60	32.20			
SLEDAI-2K	3	2	3	2	2
PhGA	50	50	50	25	25
Prednisolone dose (mg/day)	10	10	10	10	10
Anti-dsDNA	0	0	0	0	0
C3 (g/l)	0.52	0.61	0.47	0.60	0.74
C4 (g/l)	0.03	0.05	0.05	0.07	0.13
Adverse event	Oral infection	None	None	None	None

Belimumab concentrations, clinical activity, serological activity and adverse events over time in the two patients who had ADA prior to belimumab initiation.

a Lower limit of detection: 15.2 AU/ml.

ADA: anti-drug antibodies; BEL: belimumab; PhGA: physician’s global assessment; SLAM-R: revised SLAM; SLEDAI-2K: SLEDAI-2000.

## Discussion

In this cohort study of patients with active SLE treated with intravenous belimumab, we observed marked variations in drug levels among participants, although the distribution remained stable across visits at the group level. Belimumab levels were associated with clinical activity but showed no clear relationship with serological activity or the occurrence of adverse events. Notably, ADA against belimumab was detected in only 2 of 100 patients, in samples taken prior to exposure to belimumab in both patients. We found no evidence of ADA formation post exposure. This suggests that immunogenicity is unlikely to have affected the effectiveness of belimumab [29] or have contributed to the occurrence of adverse events [30].

Consistent with RCT data for intravenous [31] and subcutaneous belimumab [32], as well as recent observational data for subcutaneous belimumab [19], we observed substantial interindividual variability in belimumab levels. Notably, differences in drug levels were greater than those reported for TNF inhibitors in inflammatory arthropathies [33, 34], also after exclusion of non-trough samples within 14 days post infusion. In addition to biological factors influencing pharmacokinetics identified in experimental settings, such as body weight, IgG levels and albumin levels [31, 32], real-world settings may introduce sources of variability such as individualized dosing for reasons related or unrelated to the disease.

In cross-sectional analyses, we observed moderate correlations between belimumab levels and SLEDAI-2K and PhGA at month 6 only, while longitudinal analyses showed modest associations between belimumab levels and SLEDAI-2K and SLAM-R. These discrepancies may reflect the challenges to measure disease activity in SLE, as evidenced by the moderate agreement across instruments [35], as well as statistical power issues due to data availability. Most studies linking drug levels with clinical outcomes have focused on TNF inhibitors in inflammatory arthropathies [14]. Although several studies have reported associations between drug levels and treatment response, defining an optimal therapeutic range for a biopharmaceutical remains challenging. In contrast to TNF inhibitors, the lack of a strong association between belimumab levels and clinical activity in our study may be attributed to the fact that belimumab pharmacokinetics at a population level are not affected by immunogenicity, as suggested by our findings. This observation aligns with findings for other non-immunogenic biopharmaceuticals [36, 37].

The biological effects of belimumab are well documented, particularly its beneficial effects on complement and anti-dsDNA levels shortly after treatment initiation [6–8, 10, 24, 38]. In our per-visit cross-sectional analyses, we observed a positive correlation between belimumab levels and C3 and C4 levels, as well as an inverse correlation with anti-dsDNA titres. However, in longitudinal analysis, the effect sizes were small and non-significant. Notably, the phase II RCT of belimumab revealed no clear dose-response relationship regarding biological effects across three different intravenous doses (1, 4 and 10 mg/kg) [39]. Similarly, belimumab 1 mg/kg and 10 mg/kg resulted in similar improvements in C3 and anti-dsDNA levels in the phase III BLISS-52 and BLISS-76 trials [40]. Considering that the median belimumab concentration found herein (25.8 μg/ml; molecular weight: 146 kDa) is several orders of magnitude higher than that of its target (0.8 ng/ml, previously reported in a subset of this cohort [24]; molecular weight: 17 kDa), complete neutralization of BAFF could be anticipated, reasonably even with markedly lower dosage schemes of intravenous belimumab. Further support to this notion is the lack of evidence for target-mediated disposition in belimumab pharmacokinetic studies [31, 32], suggesting that the observed range of belimumab concentrations lies on the plateau of a dose-response curve for biological effects.

Hence, a question that arises is whether lower doses than the current 10 mg/kg every fourth week may suffice to maintain clinical and serological benefits. This hypothesis is supported by observations from phase III RCTs demonstrating a pronounced protective effect of belimumab 1 mg/kg against renal flares [41, 42], as well as observational data showing adequate disease control after 1 year in patients with low disease activity who underwent belimumab dose reductions [43]. Results from an RCT evaluating the efficacy of a fixed, lower dose of intravenous belimumab (120 mg every fourth week) are awaited to provide further insight [44]. On the contrary, the high doses may be the reason underlying the lack of immunogenicity, which in turn may contribute to the good safety profile of belimumab [30, 45]. However, this may also have untoward consequences for the B cell equilibrium as speculated after observations of *de novo* renal flares [29, 42] along with pronounced reductions in serum levels of interleukin 10 [46, 47].

Belimumab yielded no increased risk for adverse events compared with standard therapy in RCTs [30, 45]. In the present study, we observed no associations between belimumab levels and adverse events in cross-sectional or longitudinal analyses. Importantly, none of the patients who discontinued treatment due to adverse events had developed ADA. The two patients with detectable ADA experienced only mild to moderate adverse events, which however are unlikely to be related to the presence of ADA. Taken together, belimumab levels and ADA do not appear to be informative for identifying patients at risk for adverse events.

Pre-existing, naturally occurring ADA against belimumab were detected in 2 out of 100 patients: one exhibited transient ADA only before treatment initiation while the other had detectable ADA in both available samples (baseline and month 3). Although belimumab levels were low in the two patients with detectable pre-existing ADA, there was no clear impact on treatment effectiveness or safety. While ADA prior to exposure to belimumab may be naturally occurring anti-idiotype antibodies or due to cross-reactivity [48], the exact reasons underlying their development and clinical relevance remain uncertain.

Apart from pre-existing ADA, we did not observe immunogenic response following exposure to belimumab. While differences in assays to determine ADA exist, our findings align with the low immunogenicity in the pivotal belimumab trials, where ADA was observed in only 1.4% of patients [31]. These observations contrast with data on chimeric biologics such as rituximab, where immunogenicity occurs frequently (in up to 65% of patients) and affects treatment effectiveness and safety [13, 49], but also with data on human biologics such as adalimumab and golimumab, where ADA formation has been reported in up to 50% and 10% of patients with inflammatory arthropathies, respectively [50]. One could expect that SLE patients would be prone to mount an immune response against belimumab, given the polyreactive nature of the disease and supported by the higher prevalence of ADA against rituximab in SLE compared with ANCA-associated vasculitis [49]. Potential reasons for the lack of immunogenicity observed in this study include the higher belimumab dosing compared with other biologics administered via infusions, the manufacturing process (including molecular characteristics of belimumab and the lack of protein aggregates) and the concomitant use of other immunosuppressants, which has been shown to prevent the formation of ADA across different diseases [50]. Overall, our data suggest that lack of belimumab efficacy and occurrence of adverse events are unlikely due to immunogenicity.

A major strength of this study is the prospective, scheduled and consistent data collection across centres over multiple time points. Sampling was designed to occur before infusion, denoting trough levels of belimumab, and instances in which this condition was not met were excluded from the analysis of drug levels, reducing this source of variability. However, it is important to acknowledge that the results of this investigation may not be directly extrapolated to subcutaneous belimumab due to differences in pharmacokinetics between the two pharmaceutical forms [31, 32], along with anticipated logistic challenges to collect trough samples of subcutaneous belimumab.

In conclusion, belimumab yielded no immunogenicity in a setting of SLE patients from three Swedish tertiary referral centres. Belimumab levels varied widely across patients and were associated with clinical activity but not with serological activity or adverse events. Investigating clinical and immunological effects, as well as the immunogenicity, with lower doses of intravenous belimumab could provide valuable insights towards optimization of treatment regimens.

## Supplementary Material

keaf128_Supplementary_Data

## Data Availability

Data are available upon reasonable request.
