# Supplementary material for: Belimumab concentrations and immunogenicity in relation to drug effectiveness and safety in SLE within a Swedish real-world setting
Source: Rheumatology (Oxford). 2025 Mar 3;64(6):3797–805. doi: 10.1093/rheumatology/keaf128 (PMC12107032; doi:10.1093/rheumatology/keaf128)
Supplement: keaf128_Supplementary_Data [file keaf128_supplementary_data.zip › keaf128_Supplementary_Data/rhe-24-2929-File005.pdf]

Supplementary Table S1. Treatment-emergent adverse events during follow-up.

Adverse event	Frequency
Any adverse event	53
Patients with ≥1 adverse event	36
Malignancy	2
Infections	20
Erysipelas	1
Herpes zoster	2
Oral infection	1
Osteitis	1
Pneumonia	6
Upper respiratory tract infection	8
Urinary tract infection	1
Treatment-emergent adverse events	29
Anxiety	1
Headache	9
Infusion reaction	1
Insomnia	1
Lichen planus (oral)	1
Malaise	10
Nausea	2
Oropharyngeal pain	3
Pruritus	1
Laboratory abnormalities	2
Lymphopenia	1
Rhabdomyolysis	1
