# Supplementary material for: Belimumab concentrations and immunogenicity in relation to drug effectiveness and safety in SLE within a Swedish real-world setting
Source: Rheumatology (Oxford). 2025 Mar 3;64(6):3797–805. doi: 10.1093/rheumatology/keaf128 (PMC12107032; doi:10.1093/rheumatology/keaf128)
Supplement: keaf128_Supplementary_Data [file keaf128_supplementary_data.zip › keaf128_Supplementary_Data/rhe-24-2929-File006.pdf]

Supplementary Figure S1. Flow diagram of participant disposition for the ADA and belimumab concentration analyses.

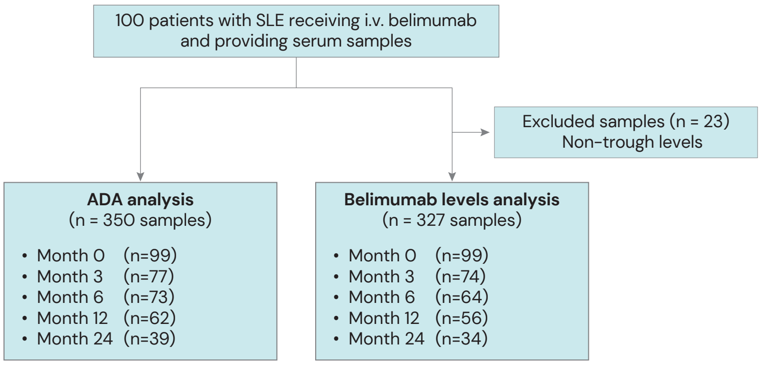

ADA, anti-drug antibody.
