# Supplementary material for: Belimumab concentrations and immunogenicity in relation to drug effectiveness and safety in SLE within a Swedish real-world setting
Source: Rheumatology (Oxford). 2025 Mar 3;64(6):3797–805. doi: 10.1093/rheumatology/keaf128 (PMC12107032; doi:10.1093/rheumatology/keaf128)
Supplement: keaf128_Supplementary_Data [file keaf128_supplementary_data.zip › keaf128_Supplementary_Data/rhe-24-2929-File008.pdf]

Supplementary Figure S3. Comparisons of belimumab drug levels by serological activity throughout follow-up.

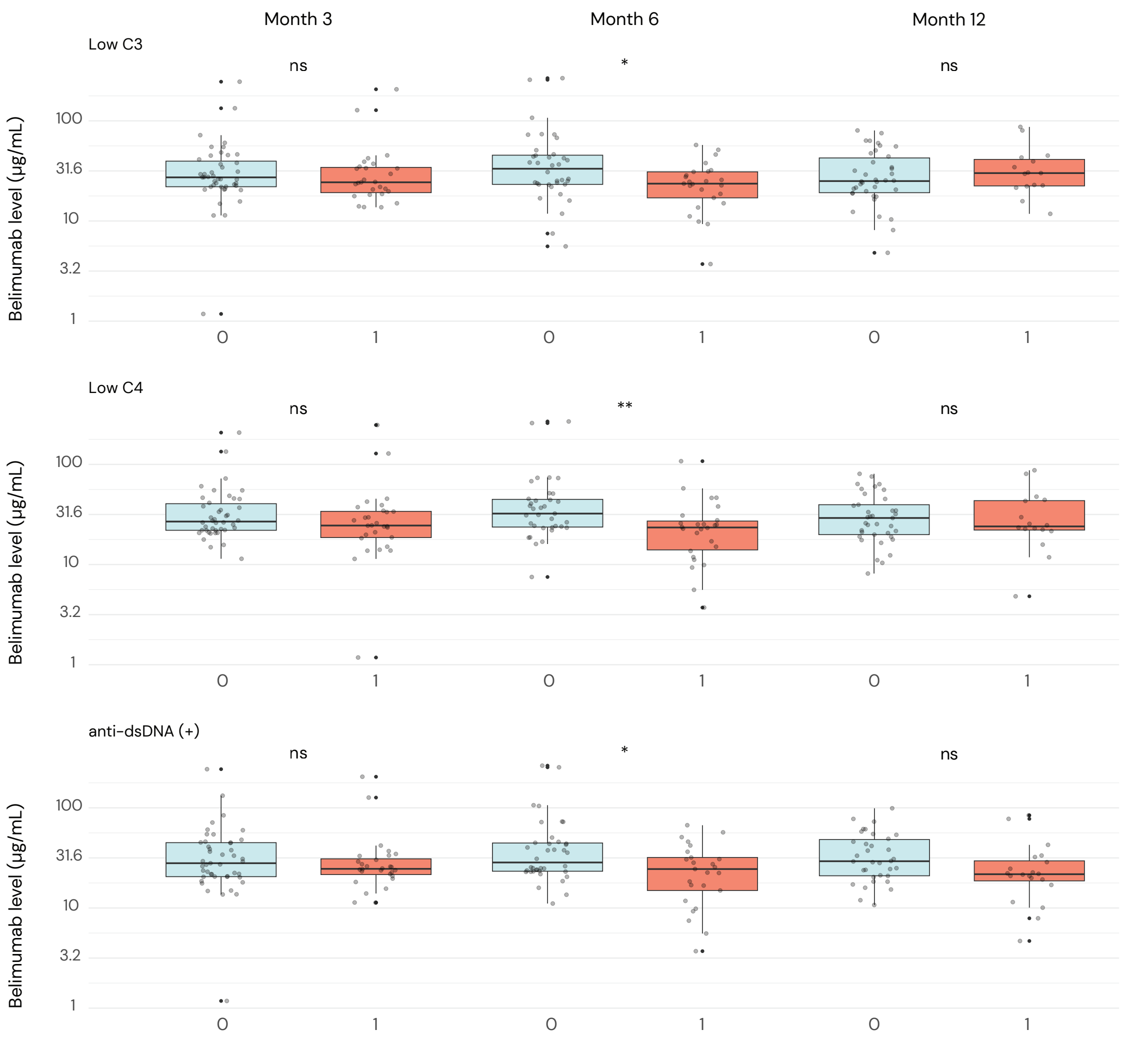

Distribution of serum belimumab levels at each visit stratified by serological activity. Grey circles denote individual measurements, while boxplots represent percentiles 10th, 25th, 50th, 75th, and 90th. Light blue denotes normal/high complement levels or anti-dsDNA negativity, while orange denotes low complement levels or anti-dsDNA positivity.
