# Supplementary figures and images for: Belimumab concentrations and immunogenicity in relation to drug effectiveness and safety in SLE within a Swedish real-world setting
Source: Rheumatology (Oxford). 2025 Mar 3;64(6):3797–805. doi: 10.1093/rheumatology/keaf128 (PMC12107032; doi:10.1093/rheumatology/keaf128)

**Supplementary Figure S2. Proportions of patients remaining on therapy during follow-up.**

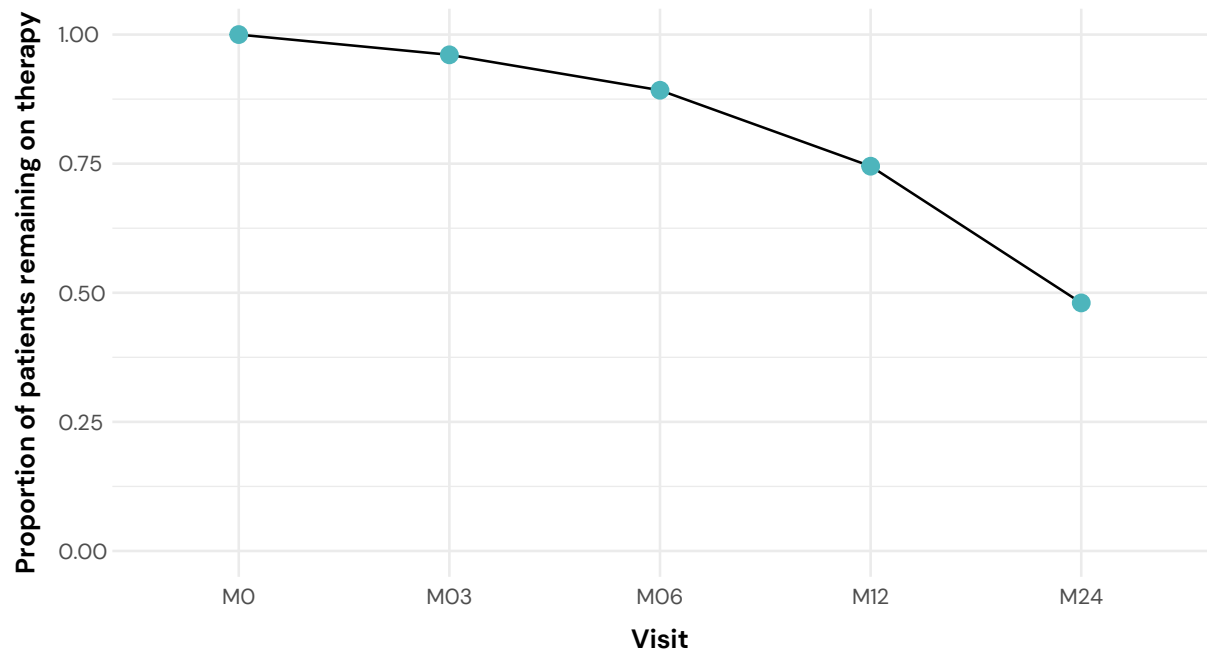

Supplement: keaf128_Supplementary_Data [file keaf128_supplementary_data.zip › keaf128_Supplementary_Data/rhe-24-2929-File007.pdf]
